# EMERGENCY PREPAREDNESS COMMUNICATION PREFERENCES OF OLDER IMMIGRANTS AND REFUGEES

**DOI:** 10.1093/geroni/igae098.0768

**Published:** 2024-12-31

**Authors:** Holly Dabelko-Schoeny, Smitha Rao, Marisa Sheldon, Anthony Traver, Fiona Doherty, Jhuma Acharya

**Affiliations:** The Ohio State University, Columbus, Ohio, United States; The Ohio State University, Columbus, Ohio, United States; The Ohio State University, Age-Friendly Innovation Center, Columbus, Ohio, United States; The Ohio State University, Columbus, Ohio, United States; The Ohio State University, Columbus, Ohio, United States; The Ohio State University, Columbus, Ohio, United States

## Abstract

Older immigrants and refugees in the U.S. face unique challenges with transportation, service access, and social integration, all critical risk factors for negative outcomes from extreme weather. This study aimed to: (1) Identify where older immigrants and refugees get information about weather-related emergencies; (2) Explore the role trusted information sources play in weather-related emergency-preparedness; and (3) Understand what supports older immigrants and refugees need to prepare for weather-related emergencies. Eleven focus groups were conducted (in English, Nepali, Tigrinya, Kinyarwanda, Somali, Amharic, Mandarin Chinese, Russian, and Khmer) with adults aged 62 and older (N = 56) in a large metropolitan Midwestern U.S. city. Most participants were immigrants and refugees, had annual incomes below $15,000, and lived alone. Preliminary analyses reveal television, radio, phone alerts, and school notifications were predominant information sources. Participants emphasized the importance of receiving communication in their native language, translation services, and simple visuals conveying preparedness information. Participants stressed the importance of reliable information sources and personal judgment in determining information trustworthiness. When asked about motivation to prepare for extreme weather, participants underscored the desire to avoid getting hurt or sick. Specifically, participants identified the need for better access to air quality information during pollution events. Participants suggested the government provide safe spaces for those without secure homes. Participants also emphasized the importance of home visits to address isolation and assistance with medication pick-up during emergencies. Findings lend insights into unique communication needs and priorities among older refugees and immigrants related to increasing extreme weather events.

